# Mobilisation and Bioavailability of Arsenic Around Mesothermal Gold Deposits in a Semiarid Environment, Otago, New Zealand

**DOI:** 10.1100/tsw.2002.101

**Published:** 2002-02-05

**Authors:** D. Craw, L. Pacheco

**Affiliations:** Geology Department and Environmental Science Programme, University of Otago, P.O. Box 56, Dunedin, New Zealand

**Keywords:** arsenic, arsenopyrite, gold mine, Otago Schist, arsenic speciation, arsenic in plants, groundwater

## Abstract

Arsenopyrite (FeAsS) is the principal arsenic (As) mineral in mineralised mesothermal veins (typically 5,000 mg/kg As) in southeastern New Zealand. Groundwater in contact with arsenopyrite-bearing rocks has elevated As concentrations (up to 0.1 mg/l). The arsenopyrite decomposes slowly on oxidation in soils and historic mine workings in a cool semiarid climate. Dissolved As is predominantly As(III) in association with arsenopyrite, but this is rapidly oxidised over days to weeks to As(V) in the vadose zone. Oxidation is facilitated by particulate Fe and/or Mn oxyhydroxides, and by bacteria in surface waters. Evaporative concentration of dissolved As(V) in the vadose zone causes precipitation of scorodite (FeAsO_4_.2H_2_O). Adsorption of As(V) to Fe oxyhydroxides in soils and groundwater pathways lowers dissolved As concentrations. Soils over mineralised veins typically have <200 mg/kg As, as most As is removed in solution on geological time scales. Most plants on the mineralised rocks and soils do not take up As, although some inedible species can fix up to 18 mg/kg As. Hence, bioavailability of As(V) is low in this environment, despite the substantial As flux.

Similar As mobility is seen in an active gold mine processing plant and tailings. Arsenopyrite dissolves more rapidly on agitation, and mine waters can have dissolved As >200 mg/l, predominantly as As(V). This dissolved As decreases in tailings waters to near 2 mg/l, mainly as As(III) when in contact with arsenopyrite. Weak oxidation of evaporatively dried tailings causes cementation with scorodite and iron oxyhydroxides, and scorodite precipitation exerts some control on dissolved As(V) concentrations. High dissolved As in mine waters is lowered by adsorption to iron oxyhydroxides, and waters discharged from the mine site have negligible dissolved As.

## INTRODUCTION

Mesothermal gold deposits consist of quartz veins crosscutting schist basement, and are typically accompanied by extensive enrichment in arsenic (As) as arsenopyrite (FeAsS). Arsenopyrite is chemically unstable in an oxidised surficial environment, and releases dissolved As on decomposition[1,2,3,4]. The fate of that As depends on the geochemistry of the environment into which it is released[2,5,6]. In high rainfall areas, As is readily removed in solution in dilute form, and may have little or no environmental impact. However in areas of low rainfall and high evaporation, the As may become enriched in soils and surface waters where it is available for uptake into the biosphere[7].

This paper describes As distribution in soils, surface waters, and shallow groundwater around mesothermal gold deposits in a cool semiarid environment. The paper addresses this mobility from two complementary approaches: natural occurrences in sites that have had little or no mining history, and a large active mine. The former sites give information on long-term processes of As mobility, whereas the latter site gives some well-constrained information on shorter-term processes. By combining information from both sources, it is possible to define some of the key features and geochemical controls on As mobility in these environments.

## GENERAL SETTING

This study examines mine sites in the Otago Schist of southern South Island of New Zealand (Fig. 1)[8]. The Otago Schist is a broad belt of sandstones and mudstones that were metamorphosed and uplifted about 140 million years ago. The rocks have been thoroughly recrystallised to a consistent mineral assemblage of quartz, albite, muscovite, chlorite, and calcite. Postuplift erosion left a broad low-relief basement surface, which was overlain by a thin veneer of tertiary sediments. Groundwater passage through these sediments caused oxidation and clay alteration of the upper 5–10 m of the underlying basement[9]. This alteration zone has now been exposed over much of the region.

Gold-bearing veins from 30 cm to 3 m wide cut the schist in scattered localities (Fig. 1). These veins contain up to 5% arsenopyrite, and arsenopyrite is also scattered through adjacent schist. The arsenopyrite is accompanied by pyrite ($FeS_2$), and gold occurs as micron-scale blebs in both sulphide minerals. Typical gold grades are between 1.5 mg/kg (bulk ore) and 20 mg/kg (vein samples). Sites around the Macraes, Barewood, and Nenthorn historic goldfields (Fig. 1) are described in this study. Historically, mining (from the 1870s to the 1930s) was small scale and principally via small underground tunnels. A large (>5 million oz) open-cut mine is currently active at Macraes, and As mobility at this site is described below.

The sites examined in this study are in the east Otago uplands, ca. 500 m above sea level. The climate is cool (mean annual temperature = 12°C) and semiarid, with precipitation between 500 and 700 mm/year. Winters have periodic, short-term (1–2 days), thin snowcover and frozen ground surface. The region is commonly swept by low humidity winds, and potential evaporation is >700 mm/year. Soils are thin (<1 m) and lithic, and vegetation is limited mainly to grasses and small shrubs. Evaporative calcite forms in soils and shallow rock fissures from soil and groundwater dissolution of basement calcite, and calcareous stalactites form in shallow mine excavations. Soil and surface water pH is generally between 7 and 8, although rainfall events contribute water with lower pH (6 to 6.5).

## METHODS

Samples of natural materials were collected from mineralised sites that have had little or no mining activity, on and near the variably oxidised exhumed basement erosion surface. Mine samples were collected from processing plant and tailings at the Macraes mine. Solid material,

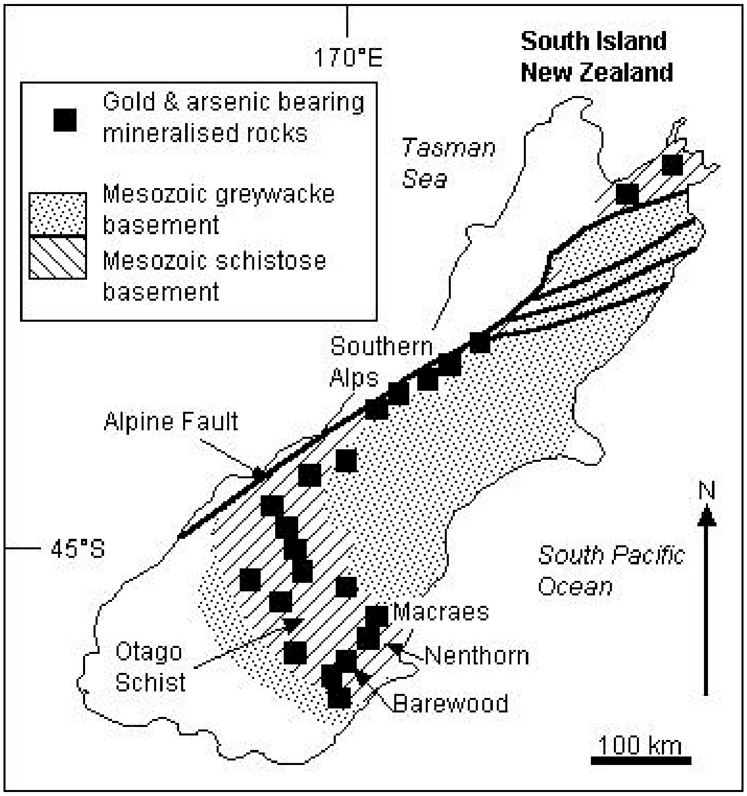

**FIGURE 1.** Geological map of the South Island of New Zealand, showing the locations of mineralised systems[8], including the sites examined in this study (Macraes, Nenthorn, Barewood) in the Otago Schist. The study area lies in a rain shadow to the east of the actively rising Southern Alps.

including soil, rock, plants, and tailings samples, was analysed by x-ray fluorescence (XRF)[10,11]. The As detection limit is ca. 2 mg/kg, with analytical error of ±2 mg/kg near the detection limit, ±5 mg/kg below 100 mg/kg, and ±10 mg/kg near 500 mg/kg. Analytical errors are ca. 1% relative in As-rich samples (>500 mg/kg).

Water samples were collected from streams, surface seeps and springs, and groundwater monitoring holes. Mine tailings waters were collected from tailings decant ponds and by centrifugation of tailings samples with immediate filtration (0.2 µm). Samples were collected in acid-washed polyethylene bottles and refrigerated until analysis.

Water samples were routinely analysed for As by atomic absorption spectrophotometry (AAS), using a graphite furnace where necessary for lower detection limits, in the Chemsearch laboratory (Chemistry Department, University of Otago), an internationally accredited laboratory. Detection limits were variably 0.5, 0.05, and 0.001 mg/l (see below), and analytical errors lay within the size of the symbols on diagrams in this paper. Inorganic As speciation was determined from analysis of total dissolved As and As(III), with As(V) determined by difference. Methylated As was not considered in this study, as these species are subordinate in this type of environment[12,13]. As(III) analysis was by flow injection AAS, using a Perkin Elmer 4100 spectrometer coupled to a continuous hydride generator (FIAS 200) and autosampler (AS 90). The method uses $NaBH_4$ as a reductant and a citrate pH buffer[14]. Results are reproducible within ca. 0.03 mg/l.

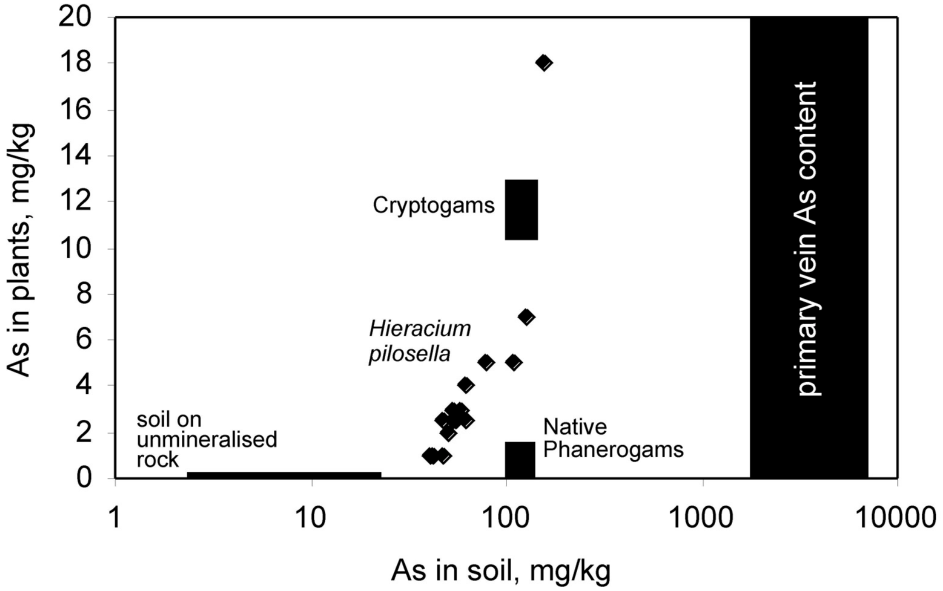

**FIGURE 2.** As contents of soils in the vicinity of a mineralised zone at Nenthorn (Fig. 1), and in plants from the same locality. As contents of background unmineralised soil (black line) and primary vein material (stippled) are indicated.

## NATURAL ARSENIC MOBILITY

### Arsenic in Solid Materials

Arsenopyrite in natural outcrops and shallow mine excavations in mineralised quartz veins is variably oxidised, due to its occurrence in the alteration zone beneath the regional erosion surface. Remnants of arsenopyrite commonly have a yellow-green tarnish or coating of scorodite ($Fe^{(III)}As^{(V)}O_4.2H_2O$) and brown Fe(III) oxyhydroxide from oxidation of coexisting arsenopyrite and pyrite. Fracture surfaces in these natural outcrops and shallow mine excavations commonly contain thin (micron-scale) smears of green scorodite on fracture surfaces. The scorodite is well crystallised, with grain size between 1 and 50 µm, and is readily identifiable with x-ray diffraction (XRD). Scorodite is rarely accompanied by pharmacosiderite ($KFe^{(III)}_4[As^{(V)}O_4]_3.[OH]_4.6H_2O$) that is also well crystallised and identifiable with XRD. The scorodite occurs in close proximity to smears of amorphous Fe(III) oxyhydroxide on fracture surfaces.

Soils developed over mineralised veins have As contents between 40 and 200 mg/kg, compared to soils on unmineralised rocks which have 2–20 mg/kg As (Fig. 2). Soil As levels are less than a tenth of the As levels in the primary quartz veins (Fig. 2). As is preferentially enriched in the millimetre-sized soil fraction and the <2-µm fraction compared to the rest of the soil[11].

Plants growing in the As-rich soils over mineralised quartz veins at Nenthorn are generally not enriched in As. Native phanerogams, including grasses and small shrubs, have As contents of 1–2 mg/kg (dry weight), near to the XRF detection limit (Fig. 2)[10]. Cryptogams (moss and lichen) growing on mineralised rocks have up to 13 mg/kg As (dry weight) (Fig. 2). A widespread exotic weed, *Hieracium pilosella*, has As content proportional to the As content of the soil in which it grows, up to 18 mg/kg in soil with 150 mg/kg As (Fig. 2)[10].

### Arsenic in Natural Waters

Streams and rivers in the east Otago area have dissolved As concentrations near to, or below, the routine graphite furnace AAS detection limit of 0.001 mg/l. This low level is maintained even in times of drought when stream flows are dominated by groundwater discharge from the schist

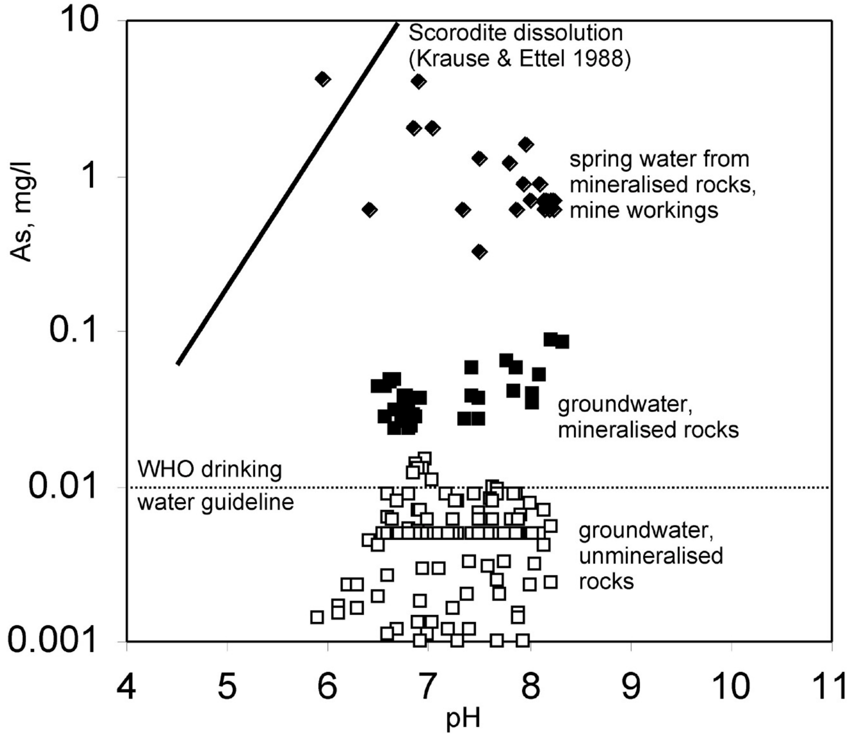

**FIGURE 3.** Dissolved As concentrations and pH of groundwaters in mineralised (arsenopyrite-bearing) and unmineralised rocks around historical mining areas in east Otago (Fig. 1), in relation to the World Health Organisation recommended drinking water guideline's maximum value for As (dotted line). The row of data at 0.005 mg/l is due to the use of that detection limit for those samples. Maximum dissolved As concentrations resulting from dissolution of scorodite (after Karause and Ettel[21]) are shown with a solid line.

basement. Groundwater in bores into fresh unmineralised basement rocks have been drilled only in the vicinity of the Macraes mine (Fig. 1), and analyses show these waters have dissolved As between 0.001 and 0.01 mg/l (Fig. 3). A cluster of data points at 0.005 mg/l in Fig. 3 represents analyses with this detection limit, and true As contents were lower. In contrast, groundwater in bores in arsenopyrite-bearing mineralised rocks near the Macraes mine have dissolved As up to 0.1 mg/l (Fig. 3). Groundwater seeps and springs in mine excavations have even higher dissolved As contents, up to 4 mg/l (Fig. 3)[11]. As enrichment of groundwater in nearby unmineralised rocks by migration of As-bearing groundwaters may be the cause of detectable dissolved As in the bores in unmineralised rocks (Fig. 3).

Groundwaters generally have pH between 6 and 8, like the surface waters (above; Fig. 3). No significant acidification of waters occurs around mineralised rocks, despite the high sulphide content, and so no acid mine drainage results from sulphide oxidation. This is because of the high calcite content of the basement rocks and soils. Oxidation of historic sulphide-rich mine tailings has resulted in a local (10-m scale) pH decrease to 5.5 at Barewood (Fig. 1), and one historic mine adit at Macraes has recorded a discharge of water with pH of 5.8 (Fig. 3).

## Arsenic Speciation of Natural Waters

One Macraes historic adit discharge water was selected for speciation study because it emerges from underground with low turbidity and is free flowing. The water deposits an amorphous Fe(III) oxyhydroxide coating on vegetation and rocks where it emerges from underground, and contains 0.85 mg/l Fe and 0.36 mg/l Mn as undeposited particulate matter that is largely removed by filtration (0.2 µm). The water also contains *Pseudomonad* bacteria, which are removed by

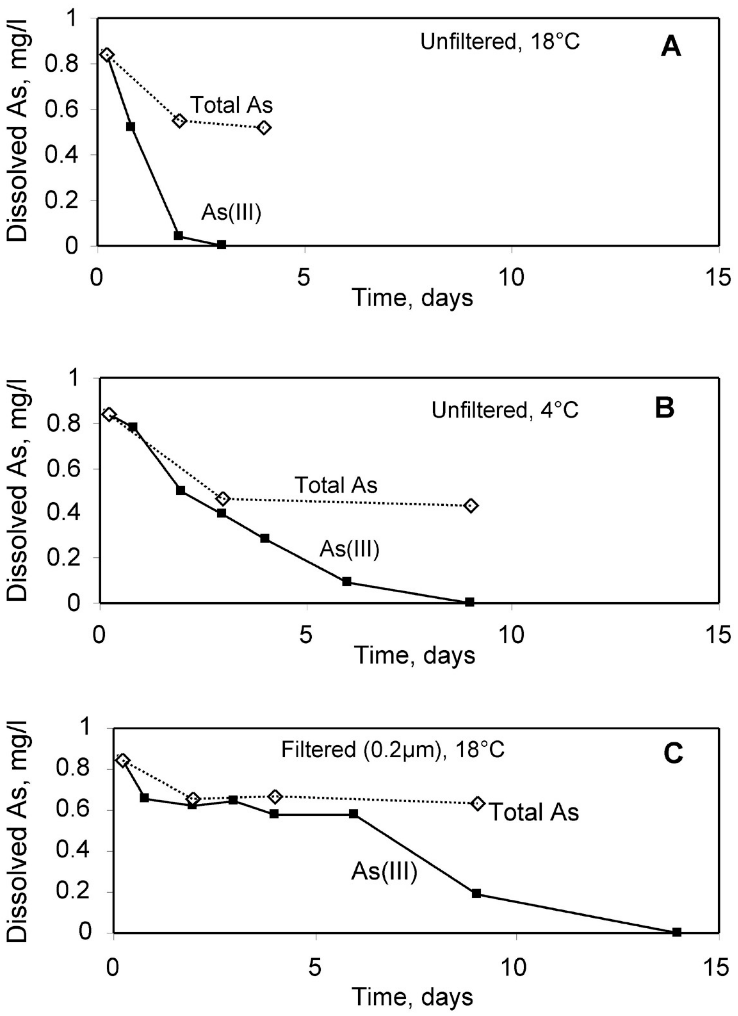

**FIGURE 4.** Experimental oxidation of As(III) with time in spring water from a Macraes historical adit: (A) unfiltered water, as collected, kept at 18°C; (B) unfiltered water kept at 4°C; (C) same water, filtered (0.2 µm) and kept at 18°C.

filtration. The emerging water had ca. 0.8 mg/l dissolved As when it was sampled for this study. This dissolved As was essentially all As(III) in samples obtained at the emergence point. From that point, the water passes through 15 m of low gradient wetland before discharging to a stream. Water sampled at the wetland discharge point had 0.3 mg/l dissolved As, essentially all of which was As(V). Some process of As extraction and oxidation occurs within the wetland segment.

To investigate further the nature and rate of the As extraction and oxidation, some laboratory experiments were conducted on water collected at the emergence point at the adit entrance. Samples of unfiltered water were kept at 4 and 18°C, and were analysed for total dissolved As and As(III) until all As was oxidised. The results of these experiments (Fig. 4A and 4B) show that complete oxidation occurs over less than 4 days at the higher temperature, and over less than 10 days at the lower temperature. Total dissolved As rapidly decreased by 25–30%, then the rate of decrease slowed in both experiments (Fig. 4A and 4B). A filtered sample (0.2 µm) kept at 18°C showed less decrease in total dissolved As than the unfiltered samples, and a slower rate of oxidation, but oxidation was complete in less than 15 days (Fig. 4C). A control sample of 1 mg/l As(III) in Milli-Q water at 18°C was analysed after the same time periods, and no decrease in As(III) was noted over 15 days.

## MINE PROCESS WATERS

The active Macraes mine provides an ideal opportunity to examine As mobility in a wide variety of environments. The mine produces large volumes of low-grade gold ore with scattered sulphide contents (ca. 1%). This material is crushed to sand (50 µm), and the sulphides are concentrated by flotation, then reground to silt or mud grain size (ca. 15 µm), before passing through the cyanidation plant for gold extraction at a pH near 10. Incipient oxidation with oxygen gas enhances gold recovery, and was used at times, but arsenopyrite passed through the processing plant largely unscathed. Tailings are accumulated in impoundments as sulphide concentrates or remixed with the bulk of the crushed rock, and process water which forms small lakes on the tailings (decant ponds) is recycled through the plant. Currently, pressure oxidation technology is being used, and all sulphides are decomposed. For this study, we present data gathered before pressure oxidation started, when arsenopyrite persisted through to the tailings.

### Arsenic Mobilisation from Arsenopyrite

Arsenopyrite is liberated from the rock in the mine processing plant, and is therefore in contact with water through the plant and in the tailings. Mobilisation of As into solution from this arsenopyrite was monitored from its entry into the cyanidation plant to its discharge into a tailings impoundment, and pore waters in tailings were examined. Water was analysed at four sites within the cyanidation process during a period in which oxygen was being used, and when oxygen was not being used. In the oxidised situation, dissolved As rose steadily to a maximum of 400 mg/l (pH = 10.4), then decreased to discharge at 260 mg/l (Fig. 5A) into concentrate tailings impoundment. The decant pond on the tailings had 160 and 260 mg/l dissolved As in two samples collected at the same time but 200 m apart, with pH near 10 (Fig. 6). Decant pond As contents can vary widely in space and time (Fig. 6), and the pond is apparently poorly mixed with respect to As contents. When oxygen was not being used in the processing plant, dissolved As levels were lower, reaching a maximum of 200 mg/l, but discharging at 46 mg/l (Fig. 5B).

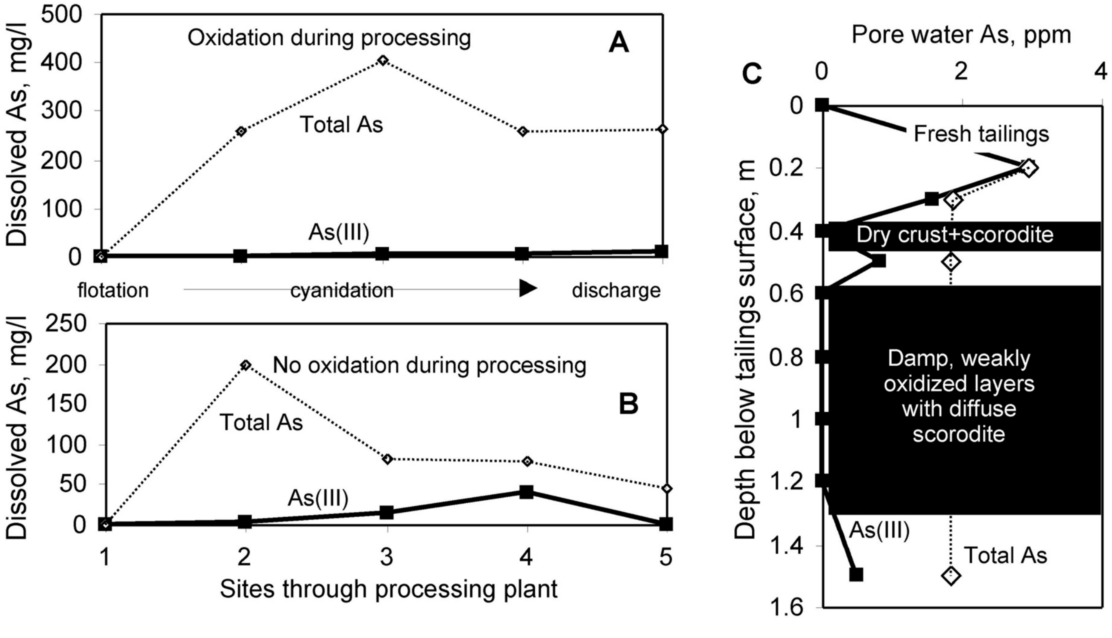

**FIGURE 5.** Inorganic speciation of dissolved As in the Macraes mine processing plant and tailings: (A) samples through the processing plant in which the system is oxidised with oxygen gas to enhance Au recovery; (B) samples through the processing plant without oxidation; (C) profile through concentrate tailings rich in arsenopyrite (see text for description).

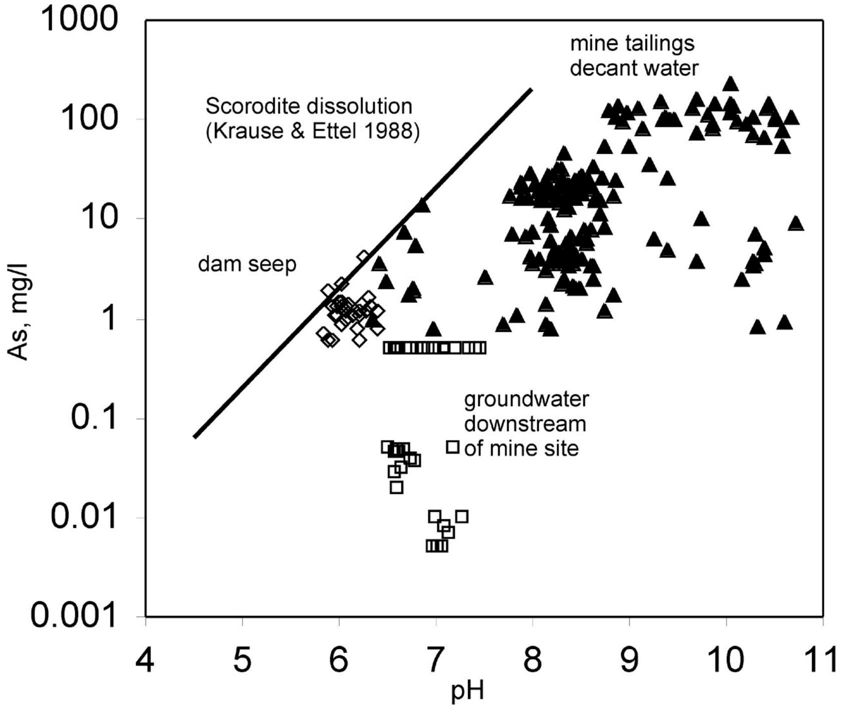

**FIGURE 6.** Dissolved As concentrations and pH (cf. Fig. 3) of mine waters at the active Macraes mine (Fig. 1), and groundwater immediately downstream in which a plume of mine water can be detected. Row of data at 0.5 mg/l is due to the use of that detection limit for those samples. Maximum dissolved As concentrations resulting from dissolution of scorodite (after Krause and Ettel[21]) is shown with a solid line.

Concentrate tailings examined for this study had a thin (ca. 30 cm) layer of fresh tailings on 5- to 10-year-old tailings that were weakly oxidised near the surface[15]. Concentrate tailings in the intervening years were mixed with flotation tailings (see below). Pore waters in fresh concentrate tailings have only 2–3 mg/l dissolved As (Fig. 5C). It was not possible to extract pore waters from most of the older tailings, which were merely moist and have some secondary cementation by Fe(III) oxyhydroxides and scorodite[15]. However, soft, wet unoxidised layers do occur, and pore waters extracted from two of these have 2 mg/l dissolved As (Fig. 5C).

There are large differences in dissolved As content between concentrate tailings discharge water (Fig. 5A and 5B) and pore waters in the tailings (Fig. 5C). Therefore, some experiments were conducted on tailings to see how much As could be easily leached. One gram (1 g) of each tailings sample was left in 10-ml distilled water for 24 h, and then the water was extracted, filtered, and analysed for dissolved As. The results of the leaching experiments showed that up to 13 mg/l dissolved As can occur in the leaching water, but that most tailings yielded leach waters with only 2–8 mg/l dissolved As (Fig. 7).

Arsenopyrite-bearing mixed tailings also have ca. 2 mg/l dissolved As in pore waters, despite higher As contents (commonly 20 mg/l) in decant pond water at a pH near 8 (Fig. 6). Similar leaching experiments were conducted on mixed tailings samples collected from cores, with similar results (mainly 2–8 mg/l leached As) to the concentrate tailings (Fig. 7). There was no clear correlation between the amount of As in the sample and the amount of leached As for the tailings (Fig. 7).

As speciation analysis of processing plant waters showed that As(III) forms a negligible component of the dissolved As during oxidative processing (Fig. 5A). As(III) can be up to 50% of the dissolved As during nonoxidative processing, although there was negligible As(III) at the discharge point (Fig. 5B) and there is no detectable As(III) in the decant pond waters. However,

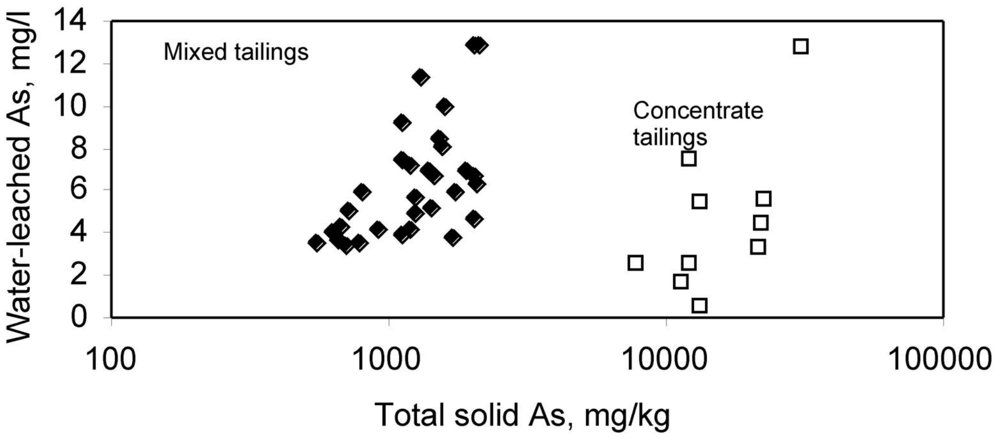

**FIGURE 7.** Concentrations of dissolved As leached with distilled water from Macraes mine tailings samples, in relation to total solid As concentrations in mixed tailings (black squares) and concentrate tailings (open squares).

fresh concentrate tailings have essentially all dissolved As as As(III) (Fig. 5C). Older wet tailings have detectable, but small, proportions of As(III) (20–30%; Fig. 5C). The weakly oxidised older tailings contain scorodite and are presumed to have all dissolved As as As(V).

Data were presented above for mine process waters with highly elevated dissolved As contents, generally between 2 and 200 mg/l (Fig. 6), and locally even higher. These waters are recycled where possible, but some water losses occur below impoundment dams[16] and a seep from a dam structure. A low-volume plume of mine waters is detected in groundwater wells 50 m downstream from the main tailings dam[16]. It is notable that the high dissolved As contents of the mine site are lowered to less than 2 mg/l in the dam seep (Fig. 6), and do not appear in the downstream groundwater that has dissolved As levels typical of mineralised rocks (Fig. 3 and 6).

## DISCUSSION AND CONCLUSIONS

### Dissolution of Arsenopyrite

Arsenopyrite is thermodynamically unstable under surface environmental conditions[2], and decomposes in water to release dissolved As[1,4]. However, this decomposition must be slow, as water in contact with arsenopyrite in the concentrate tailings has less than 3 mg/l dissolved As (Fig. 5C) after up to 5 years. Springs from arsenopyrite-bearing rocks can have dissolved As up to ca. 4 mg/l, but generally are below 1 mg/l (Fig. 3). Slow-moving groundwater in arsenopyrite-bearing rocks has even lower dissolved As contents (0.1–0.01 mg/l; Fig. 3). Hence, despite its thermodynamic instability, arsenopyrite remains relatively unaltered in east Otago water-saturated environments. This conclusion is supported by observations of 29,000-year-old detrital arsenopyrite in thin (<5-m thick) sediments near the Macraes mine[16].

Arsenopyrite decomposes more rapidly where it is exposed to both oxygen and water[3]. Short-term decomposition occurs in the Macraes mine processing plant, where dissolved As concentrations can reach over 400 mg/l (Fig. 5A). This high level of dissolved As is a result of oxidation and agitation of the mineral material as it passes through the plant system. Exposure of arsenopyrite in the vadose zone of soils results in long-term decomposition and leaching of As, so that originally high solid As levels (>1000 mg/kg) are lowered by at least an order of magnitude (Fig. 2).

## Arsenic Speciation

Observations described above suggest that water in contact with arsenopyrite has dissolved As as As(III). This is most dramatically demonstrated in the fresh Macraes concentrate tailings, where process water with negligible As(III) evolves to pore water with only As(III) on a time scale of weeks (Fig. 5C). Even 5-year-old tailings that are undergoing weak oxidation have significant As(III) preserved (Fig. 5C). Spring water emanating from an historic mine adit in arsenopyrite-bearing rocks has all dissolved As as As(III) (Fig. 4).

As(III) is rapidly oxidised to As(V) in the surficial environment[6,12]. This oxidation can occur on a time scale of hours in the Macraes mine processing plant (Fig. 5A), although the oxidation is inhibited when oxygen gas is not involved (Fig. 5B). Oxidation of As(III) occurs on a time scale of days or weeks in the adit spring water (Fig. 4A, 4B, and 4C). Oxidation is slower at lower temperatures (Fig. 4B, representative of winter) than at higher temperatures (Fig. 4A, representative of summer). Filtration of the water slowed down the As(III) oxidation by a few days (Fig. 4C). Oxidation of As(III) is probably mediated by microparticulate iron and/or manganese oxyhydroxides[17,18], some of which may pass through even 0.2 µm filters[19]. The *Pseudomonad* bacteria probably contribute to As(III) oxidation[20], but bacteria were absent from the filtered experiment (Fig. 4C).

## Fixation of As(V)

As(V) is highly mobile in the surficial environment at circumneutral pH[2]. However, fixation of As(V) can occur by two principal, and related, mechanisms: scorodite formation and adsorption. Formation of scorodite (or other minerals) is the most visible way in which As(V) is fixed in the east Otago environment. Scorodite forms in the vadose zone of soils and mine tailings (above), especially in dry rocks and tailings where evaporative concentration of As-bearing waters has occurred. The concentration of As(V) required to precipitate scorodite is strongly dependent on pH, and rises with rising pH (Fig. 3 and 6)[21]. The dissolved As content of spring waters from mine workings may be limited by scorodite formation (Fig. 3). Likewise, circumneutral pH mine waters at the Macraes mine may have dissolved As limited by scorodite formation (Fig. 6).

Adsorption of As onto mineral surfaces, especially Fe(III) oxyhydroxides, is a widespread phenomenon[5,19,22,23], and results in at least temporary fixation of As. This process is more pronounced for As(V) than for As(III)[6,24]. Some of the As adsorbed to Fe(III) oxyhydroxides is strongly bound and has a scorodite-like structure[25]. The large drop in dissolved As between the concentrate tailings decant pond (As >100 mg/l) and tailings pore waters (As <3 mg/l) is almost certainly due to adsorption of As onto Fe(III) oxyhydroxides, which are suspended in the decant waters[15]. Likewise, extraction of As from mine waters before discharge into the environment (Fig. 6) is due to adsorption by Fe(III) oxyhydroxides[25]. Leached As from tailings (Fig. 7) may be remobilisation of this adsorbed As, renewed dissolution of arsenopyrite, or a combination of both processes. High As contents of the finest fraction (<2 µm) of soils over mineralised rocks (above) is a result of adsorption of As on to fine-grained Fe(III) oxyhydroxides[11]. The same process is probably occurring in wetlands where As-bearing spring waters emerge from historic mine workings. Total dissolved As in such waters decreases with time (Fig. 4A, 4B, and 4C) because of adsorption to microparticulate metal oxyhydroxides.

## Bioavailibility of As

As(III), the more toxic form of As, is not bioavailable in surficial environments in east Otago because it is oxidised to As(V) on a time scale of hours or days. As(III) does persist in pore waters in arsenopyrite-bearing concentrate tailings at the Macraes mine, but these pore waters are effectively sealed off from the surficial environment by the low permeability of the tailings[15].

Elevated As(V) is widespread in groundwater seeps, mine waters, and soils in east Otago, but uptake is low in plants, except for an unpalatable exotic weed, *Hieracium pilosella*. The As(V) is fixed in solid form temporarily by adsorption, and by evaporative deposition of scorodite. This As is progressively leached by percolating waters, and removed from the soil. Dissolved As from dissolution of arsenopyrite and from leaching of soil As is flushed into rivers, where the As load is diluted to below the World Health Organisation's recommended drinking water level. There is a large flux of As through the east Otago environment, with localised temporary enrichment, but this large flux has essentially no biological impact.

## ACKNOWLEDGEMENTS

This paper is an outgrowth of a paper presented at the 6th International Conference on the Biogeochemistry of Trace Elements, Guelph, Ontario, Canada, 29 July–2 August, 2001. Financial support was provided by the New Zealand Public Good Science Fund and the University of Otago. Jenny Webster-Brown and Kerry Webster assisted with analytical techniques. Debra Chappell assisted with some of the sampling, and kindly assisted in identification of bacteria. Analyses of solid materials and leaching experiments were conducted with the assistance and advice of Tony Reay and Damian Walls. Additional information and water analyses were provided by GRD Macraes Ltd. GRD Macraes Ltd also kindly provided access to their mine site and logistical assistance, and the help and enthusiasm of Debbie Wilson, Adrian Marin, and John Bywater was especially appreciated.
